# Factors explaining the gender disparity in lipid-lowering treatment goal attainment rate in Chinese patients with statin therapy

**DOI:** 10.1186/1476-511X-11-59

**Published:** 2012-05-29

**Authors:** Rui Zhang, Liancheng Zhao, Lirong Liang, Gaoqiang Xie, Yangfeng Wu

**Affiliations:** 1 Department of Epidemiology and Biostatistics, School of Public Health, Peking University Health Science Center, Beijing, 100191, China; 2 Division for CVD Prevention and Control Network of National Center for Cardiovascular Disease Control and Research, Fuwai Hospital, Peking Union Medical College and Chinese Academy of Medical Sciences, Beijing, 100037, China; 3 Beijing Institute of Respiratory Medicine, Beijing Chao-yang Hospital, Capital Medical University, Beijing, 100020, China; 4 Peking University Clinical Research Institute, Beijing, 100191, China; 5 The George Institute for Global Health China, Beijing, 100088, China

**Keywords:** Dyslipidemia, Lipid-lowering treatment, Goal attainment, Gender disparity, Patient

## Abstract

**Background:**

The lipid-lowering treatment goal attainment rate is lower for women than for men among Chinese patients, but the reasons for this disparity have not been fully explored yet.

**Objectives:**

To elucidate the potential factors and the significance of their contributions towards the observed discrepancy in lipid-lowering treatment goal attainment rates between Chinese women and men.

**Methods:**

We used data from 1808 patients from 21 tertiary and 6 secondary hospitals in China who received and maintained statin therapy treatment for at least 2 months. Lipid-lowering treatment goal attainment was defined as low-density lipoprotein cholesterol (LDL- C) reaching the treatment targets recommended by the Chinese Guidelines on Prevention and Control of Dyslipidemia in Adults. Logistic Regression was used to explore possible factors associated with gender disparity in goal attainment rates, and to what extent each factor contributes.

**Results:**

A total of 674 women and 1134 men were enrolled in the study. Women had a significantly lower LDL-C goal attainment rate than that of men (46.0% vs 53.8%, P = 0.002), particularly in high and very high CVD risk groups. Among high and very high risk patients, approximately 35%, 7%, 5%, and 5% of gender disparity in LDL-C goal attainment rate was attributable to the gender difference in baseline LDL-C level, cardiovascular co-morbidities and associated risk factors, socioeconomic status, and the dosage of statin treatment, respectively. Approximately 50% of the gender disparity remained unexplained by these factors.

**Conclusions:**

Although nearly half of the gender disparity in lipid-lowering treatment goal attainment rate can be explained by the gender differences in baseline lipid level, socioeconomic status, cardiovascular co-morbidities and associated risk factors, and the dosage of statin in high and very high CVD risk patients, the other half of the gender disparity remains unexplained and requires further study to fully understand what other factors are at play.

## Background

Cardiovascular diseases (CVD) such as coronary heart disease (CHD) and stroke are the leading causes of death for both men and women in China [1,2]. Dyslipidemia management is one of the most important strategies for the prevention of CVD and has been shown to reduce cardiovascular risk in both men and women [3-6]. However, previous studies suggest that only a small proportion of women with dyslipidemia achieve optimal lipid levels [7,8], and that even when their access to lipid-lowering treatment is similar to that of men, women are less likely to reach their lipid treatment goal than men, especially in high risk groups [9-12]. The EUROASPIRE survey showed that LDL-C treatment goal attainment rates were approximately 20% lower in women than men [11]. A similar finding has been demonstrated in L-TAP surveys [10,12]. Although many researchers have observed gender disparities in LDL-C goal attainment rate in patients receiving lipid-lowering treatment, few have clarified on the reasons for this gender disparity.

The purpose of this study is to confirm a gender disparity in lipid-lowering treatment goal attainment rate among Chinese patients and explore the potential factors that might help explain the gender imbalance using data from the Second Multi-Center Study of Clinical Management of Dyslipidemia in China.

## Methods

### Study participants

The Second Multi-Center Study of Clinical Management of Dyslipidemia in China was conducted from January 1 until May 31 in 2006. Data was collected from 21 tertiary hospitals and 6 secondary hospitals in 12 cities across mainland China. The design of this study has been described in detail elsewhere [13,14]. Briefly, trained and certified research physicians recruited dyslipidemic patients consecutively if they met the following criteria: (1) they were ≥ 20 years old; (2) they initiated their lipid-lowering drug treatment in the period between January 1, 2004 and February 28, 2006; (3) The lipid-lowering drug should remain unchanged at its initial drug dosage for at least two months; (4) serum lipid test results must be available both within four weeks before the commencement of the study and within 2 weeks after the stop/change of the lipid-lowering treatment; and (5) no history of lipid-lowering drugs use within six months prior to the commencement of the current lipid-lowering drugs. Patients were excluded if they met one or more of the following conditions: hypothyroidism, nephritic syndrome, trauma, pregnancy, breast-feeding, or treatment with a lipid-altering drug or device being investigated during the study period. Of the 2306 patients using lipid-lowering therapy initially selected, 2237 patients met the above inclusion criteria.

Among 2237 patients, 1808 participants were treated with statin therapy and were analyzed in our study. The patients treated with other drugs who were excluded from the study analysis were slightly younger (60.4 ± 11.9 vs 62.5 ± 11.3, P = 0.004) with lower LDL-C levels (2.82 ± 0.99 vs 3.24 ± 1.02, P = 0.001) at baseline, and the LDL-C treatment goal attainment rate was lower (43.0% vs 50.9%, P = 0.001) compared to patients who participated in the study; however, the proportion of women who were excluded and included was similar (37.4% vs 37.3%, P = 0.507).

The study was conducted in accordance with the Declaration of Helsinki. All patients gave written informed consent, and the study was approved by the institutional review board of the Cardiovascular Institute and Fuwai Hospital in Beijing, China.

### Data collection

The medical charts of eligible patients were reviewed by centrally trained and certified research physicians. Requested information was collected and transcribed to a standard form. The baseline information was collected when drug therapy was initiated in patients during the period from January 2004 to February 2006. Collected information included: (1) demographic information such as age, gender, educational level, occupation, coverage rate of medical insurance, hospital level care; (2) co-morbidities including CHD (acute myocardial infarction, coronary intervention, or coronary artery bypass grafting), ischemic stroke (cerebral thrombosis, cerebral embolism), hypertension status (defined as systolic blood pressure ≥ 140 mm Hg and/or diastolic blood pressure ≥ 90 mm Hg and/or any anti-hypertensive medication), diabetes mellitus (DM, defined as fasting serum glucose ≥ 7.0 mmol/L and/or oral glucose tolerance test ≥ 11.1 mmol/L and/or glycated hemoglobin A1c ≥ 6.5% and/or lipid diabetes medication use), peripheral angiopathy diseases (PAD, including carotid artery plaque, abdominal aortic aneurysm or intermittent claudication); (3) cardiovascular risk factors including currently smoking (defined as ≥ 1 cigarette per day for at least 1 year), body mass index (BMI, defined as weight in kilograms divided by the square of height in meters), family history of premature CHD (defined as CHD before 55 years of age in male first-degree relatives or before 65 years of age in female first-degree relatives); (4) name of lipid-lowering drugs and their dosages (mg/d); (5) status of therapeutic lifestyle changes; (6) the levels of LDL-C, high-density lipoprotein cholesterol (HDL-C), total cholesterol (TC). and triglycerides (TG) before and after the drug therapy. Statin dosage was classified into equipotency dose groups according to their LDL-C lowering efficacy on a mg : mg basis [15].

### Definition of lipid-lowering treatment goal attainment rate

The lipid-lowering treatment goal attainment rate was defined as the proportion of patients achieving their LDL-C treatment goals with lipid-lowering treatment according to the Chinese Guidelines on Prevention and Treatment of Dyslipidemia in Adults [16]. Patients were classified into low, moderate, high, and very high risk groups according to the guidelines based on their history of cardiovascular diseases (CHD, ischemic stroke, PAD and DM) and other risk factors. These risk factors included older age (≥ 45 years for men, ≥ 55 years for women), currently smoking, low HDL-C level (< 1.04 mmol/L), obesity (BMI ≥ 28 kg/m^2^), and family history of premature CHD and hypertension. The LDL-C treatment goals were LDL-C < 4.14 mmol/L, < 3.37 mmol/L, < 2.59 mmol/L, and < 2.07 mmol/L for low, moderate, high, and very high risk groups, respectively.

### Statistical analysis

Continuous variables were presented as mean and standard deviation (SD) and categorical variables as proprotions. LDL-C goal attainment rate and differences in categorical variables were compared between genders using the *χ*^2^ test. Differences in continuous variables between genders were compared by the Student’s *t*-test. We used a base model where only gender was included as the independent variable to model the difference in goal attainment between men and women, as shown by the odds ratio (OR) in the logistic regression model. We then added each of those variables that showed a significant difference between men and women into the base model and calculated the percentage of difference of OR for gender after adding the variable. The percentage of difference after adding a specified variable to the model was calculated using the following formula:

(1) $$\left[\left({\text{ OR}}_{1}-{\text{ OR}}_{2}\right)/\left({\text{ OR}}_{1}-\;1.0\;\right)\right]\times \;100\%$$

Where OR_1_ represents OR of gender derived from the base model, or the model without adding the designated variable; and OR_2_ represents OR of gender after adding in the designated variable(s). A p-value less than 0.05 was accepted as statistically significant. All analyses were performed with SPSS statistical software version 13.0 (SPSS Inc., Chicago, IL, USA).

## Results

### Clinical characteristics of study population

The study population included 674 (37.3%) women and 1134 men aged between 23 and 91 years of age. Among these patients, women were slightly older, more likely to have a low level of education, more likely to be unemployed, and had a low level of medical insurance coverage compared to men. Women also had a greater prevalence of hypertension and diabetes, but had a lower prevalence of CHD, current smokers and received less treatment in tertiary hospitals compared to men. Furthermore, women had higher TC, LDL-C, HDL-C levels than men before and after statin therapy. In addition, women were more likely to be treated with a low dosage of statin (Table 1). Among the study patients, 48.6% (180/370) of women and 66.5% (547/823) of men with CHD underwent coronary intervention or coronary artery bypass grafting. Only 48 out of 1808 patients took combined treatment of statin and other lipid-lowering drugs such as fibrates, nicotinic acids, and other medications. Since the number of patients with combined drug treatment was very small (2.7%), it did not significantly affect the study results (data not shown).

**Table 1:** Clinical characteristics of the study patients according to gender

	**Women**	**Men**
**N**	674	1134
**Age**	64.1 ± 9.6^#^	61.5 ± 12.1
**Educational level**		
Primary middle school or lower	306 (45.4)	305 (26.9)
High school and middle school	160 (23.7)	313 (27.6)
Junior college or higher	208 (30.9)^#^	516 (45.5)
**Occupation**		
Administrative	145 (21.5)	471 (41.5)
Non-administrative	106 (15.7)	149 (13.0)
Unemployed	423 (62.8)^#^	514 (45.5)
**Coverage rate of medical insurance**		
0-69%	294 (43.8)	451 (39.9)
≥70%	378 (56.3)^#^	680 (60.1)
**Hospital level**		
Tertiary	510 (75.7)	961 (84.7)
Secondary	164 (24.3)^#^	173 (15.3)
**Body Mass Index (kg/m**^**2**^**)**	24.7 ± 3.6	25.0 ± 3.1
**Co-morbidities**		
CHD	370 (54.9)^#^	823 (72.6)
PAD	48 (7.1)	82 (7.2)
Stroke	62 (9.2)	109 (9.6)
DM	219 (32.5)^#^	330 (29.1)
Hypertension	533 (79.1)^#^	818 (72.1)
**Obesity (BMI ≥ 28.0)**	113 (16.8)	162 (14.3)
**Currently smoking**	20 (3.0)^#^	309 (27.2)
**Family history of premature CHD**	44 (6.5)	60 (5.3)
**Risk stratification**		
Low	63 (9.3)	77 (6.8)
Moderate	76 (11.3)	53 (4.7)
High	300 (44.5)	391 (34.5)
Very high	235 (34.9)^#^	613 (54.1)
**The dosage of statin treatment**		
Low dose	273 (40.5)	397 (35.0)
Standard dose	342 (50.7)	628 (55.4)
High dose	59 (8.8)	109 (9.6)
**Diet modification**	574 (85.2)^#^	908 (80.1)
**Lipids before therapy**		
TC (mmol/L)	5.99 ± 1.28^#^	5.18 ± 1.25
LDL-C (mmol/L)	3.51 ± 1.03^#^	3.08 ± 0.99
HDL-C (mmol/L)	1.35 ± 0.42^#^	1.15 ± 0.35
TG (mmol/L)	2.15 ± 1.25	2.07 ± 1.61
**Lipids after therapy**		
TC (mmol/L)	4.98 ± 1.12^#^	4.39 ± 1.02
LDL-C (mmol/L)	2.75 ± 0.87^#^	2.43 ± 0.78
HDL-C (mmol/L)	1.39 ± 0.41^#^	1.18 ± 0.33
TG (mmol/L)	1.80 ± 0.93	1.71 ± 1.14

Value are mean ± SD or n (%); CHD, coronary heart disease; PAD, peripheral angiopathy disease; DM, diabetes mellitus; LDL-C, low-density lipoprotein cholesterol, HDL-C, high-density lipoprotein cholesterol; TC, total cholesterol; TG, triglycerides.

^#^*P* <0.05, compared with men.

### LDL-C goal attainment

**Figure 1:**
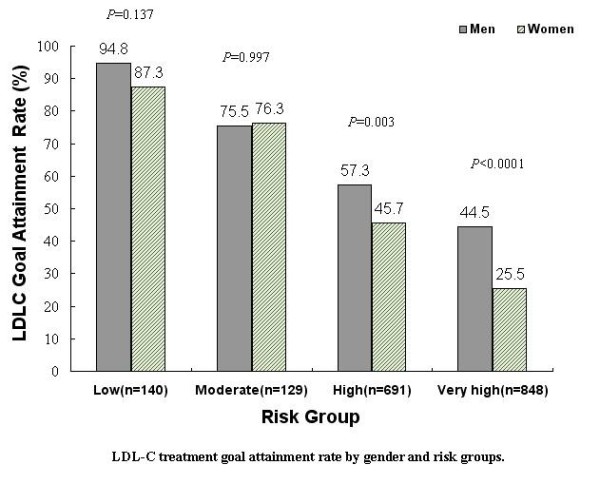
LDL-C treatment goal attainment rate by gender and risk groups.

Fewer women reached their LDL-C treatment goals than men (46.0% vs 53.8%, *P* = 0.002). As illustrated in Figure 1, this difference was observed in high and very high risk groups, where only 45.7% and 25.5% of women compared to 57.3% and 44.5% of men (*P* = 0.003 and *P* < 0.001) attained lipid-lowering treatment goals, respectively. No significant difference was found in the LDL-C goal attainment rate between genders in low and moderate risk groups.

### Potential factors for gender disparity

Table 2 shows the LDL-C goal attainment rates in women and men and the relative differences according to the characteristics of the study population. Socioeconomic status including educational levels, occupation, and coverage rate of medical insurance, cardiovascular co-morbidities and associated risk factors that include hypertension, CHD, stroke, DM, PAD, family history of premature CHD and currently smoking, hospital level care, baseline LDL-C level, and the dosage of statin treatment may be the potential factors for the observed gender disparity in LDL-C goal attainment.

**Table 2:** LDL-C goal attainment rates in women and men, and the relative differences overall and by characteristics of the study population

	**Women (F)**	**Men (M)**	Relative diff.(%) (M-W)/M*100%
**Overall**	**46.0**	**53.8**	**14.5**
**Age**			
<65	**45.5**	**54.0**	**15.7**
<65> = 65	**46.5**	**53.5**	**13.1**
**Educational level**			
<65Primary middle school or lower	**60.0**	**71.4**	**16.0**
<65High school and middle school	**42.8**	**52.4**	**18.3**
Junior college or higher	**49.8**	**56.5**	**11.9**
**Occupation**			
Administrative	**46.2**	**56.3**	**17.9**
Non-administrative	**42.5**	**53.7**	**20.9**
Unemployed	**46.8**	**51.7**	**9.5**
**Coverage rate of medical insurance**			
0-69%	**44.6**	**53.7**	**16.9**
≥70%	**46.8**	**53.8**	**13.0**
**Hospital level**			
Tertiary	**42.2**	**52.7**	**19.9**
Secondary	**57.9**	**60.1**	**3.7**
**Co-morbidities**			
CHD	**34.3**	**49.7**	**31.0**
PAD	**51.0**	**42.7**	**−19.4**
Stroke	**39.1**	**50.0**	**21.8**
DM	**31.5**	**43.9**	**28.2**
Hypertension	**44.7**	**52.8**	**15.3**
**Obesity (BMI ≥ 28.0)**			
Yes	**40.7**	**47.2**	**13.8**
No	**47.2**	**55.0**	**14.2**
**Currently smoking**			
Yes	**60.0**	**54.7**	**−9.7**
No	**45.6**	**53.5**	**14.8**
**Family history of premature CHD**			
Yes	**36.4**	**45.0**	**19.1**
No	**45.0**	**53.8**	**16.4**
**Risk stratification**			
Low	**87.3**	**94.8**	**7.9**
Moderate	**76.3**	**75.5**	**−1.1**
High	**45.7**	**57.3**	**20.2**
Very high	**25.5**	**44.5**	**42.7**
**The dosage of statin treatment**			
Low dose	**34.4**	**48.9**	**29.7**
Standard dose	**53.5**	**57.2**	**6.5**
High dose	**55.9**	**52.3**	**−6.9**
**Diet modification**			
Yes	**46.3**	**54.3**	**14.7**
No	**44.0**	**51.8**	**15.1**
**LDL-C (mmol/L) before therapy**			
<3.37	**56.4**	**60.2**	**6.3**
≥3.37	**37.7**	**42.8**	**11.9**

CHD, coronary heart disease; PAD, peripheral angiopathy disease; DM, diabetes mellitus; LDL-C, low-density lipoprotein cholesterol, HDL-C, high-density lipoprotein cholesterol; TC, total cholesterol; TG, triglycerides; BMI, body mass index.

Firstly, we used Logistic regression models to examine the independent impact of gender on LDL-C goal attainment in high and very high risk groups, since gender disparity in LDL-C goal attainment rate only existed in high and very high risk groups. The OR comparing LDL-C goal attainment in women versus men was 0.59 in high and very high risk groups in the base model (Table 3). When each potential factor was added to the base model, the OR changed from 0%-37%. Among the variables, baseline LDL-C level produced the largest change in OR of gender (36.6%), followed by cardiovascular co-morbidities and associated risk factors (7.3%), the dosage of statin (4.9%), and socioeconomic status (4.9%). Age and hospital level care led to a decrease in OR (− 2.4% and - 4.9%, respectively).

**Table 3:** Contributions of each covariate in gender difference in LDL-C goal attainment among patients with high and very high risk, single variable analysis

**Logistic regression models**	**OR of gender (95%CI)**	**Percentage of gender difference accounted**
Base model : gender (women/men)	0.59 (0.48-0.73)	-
Base model + Age	0.58 (0.47-0.72)	−2.4%
Base model + Hospital level	0.57 (0.46-0.71)	−4.9%
Base model + Socioeconomic status^*^	0.61 (0.49-0.76)	4.9%
Base model + Cardiovascular co-morbidities and risk factors^#^	0.62 (0.49-0.78)	7.3%
Base model + Dosage of statin in use	0.61 (0.49-0.76)	4.9%
Base model + Baseline LDL-C	0.74 (0.59-0.94)	36.6%

^*^ Including education level, occupation and coverage rate of medical insurance.

^#^ Including hypertension, coronary heart disease, stroke, diabetes mellitus, peripheral angiopathy disease, family history of premature CHD and current smoking.

LDL-C, low-density lipoprotein cholesterol; OR, odds ratio.

As illustrated in Table 4, when covariates such as age, socioeconomic status, cardiovascular co-morbidities and associated risk factors, hospital level care, dosage of statin treatment, and baseline LDL-C levels were added to the base model step by step, the OR of gender changed from 0.59 to 0.77. In the complete model, including the above-mentioned variables, 44% of gender disparity was accounted for and the OR of gender remained statistically significant.

**Table 4:** Contributions of each covariate in gender difference in LDL-C goal attainment among patients with high and very high risk, multiple variable analysis

**Independent variables in Logistic regression models**	**OR of gender (95%CI)**	**Percentage of gender difference accounted**
Gender (Base model)	0.59 (0.48-0.73)	-
Variables in above model + Age	0.58 (0.47-0.72)	−2.40%
Variables in above model + Socioeconomic status^*^	0.60 (0.48-0.75)	2.40%
Variables in above model + Cardiovascular comorbidities and risk factors^#^	0.63 (0.50-0.80)	9.80%
Variables in above model + Hospital level	0.62 (0.48-0.78)	7.30%
Variables in above model + dosage of statin	0.63 (0.50-0.81)	9.80%
Variables in above model + baseline LDL-C level	0.77 (0.60-0.98)	44.00%

^*^ Including education level, occupation and coverage rate of medical insurance.

^#^ Including hypertension, coronary heart disease, stroke, diabetes mellitus, peripheral angiopathy disease, family history of premature CHD and current smoking.

LDL-C, low-density lipoprotein cholesterol; OR, odds ratio.

In addition, when we repeated the same analyses in all patients, the results were similar to the results in the patients with high to very high risk. The main factors explaining gender disparity in lipid-lowering treatment goal attainment rate for all patients were also socioeconomic status, cardiovascular co-morbidities and associated risk factors, dosage of statin treatment, and baseline LDL-C levels, but these variables totally accounted for approximately 25% of gender disparity (data not shown).

## Discussion

To the best of our knowledge, this is the first study to quantitatively estimate the factors for disparity in lipid-lowering goal attainment rate between women and men receiving stable doses of statin therapy in Chinese hospitals. Our results indicate that nearly half of the gender disparity in patients with high and very high risk in lipid-lowering treatment goal attainment rate can be explained by the differences in socioeconomic status, cardiovascular co-morbidities and associated risk factors, baseline lipid level, and the dosage of statin treatment; however, the other half of the gap remains unexplained. In addition, of all the potential factors we examined, the baseline LDL-C level was the primary contributor to the gender disparity.

The current study demonstrates that the lipid-lowering treatment goal attainment rate was lower for women than for men undergoing stable lipid-lowering treatment, especially in the high and very high risk groups, even after adjusted for other covariates, in hospitals across mainland China. These findings are consistent with previous studies [9-12]. The L-TAP 2 survey included 9955 patients from 9 countries on stable lipid- lowering treatment for at least 3 months and indicated that overall goal attainment rate of patients reaching their LDL-C goal was 71.5% in women and 73.7% in men (*P* = 0.014), and the goal attainment rate in the high risk/CHD groups was 62.6% in women and 70.6% in men (*P*<0.001) [10]. Another survey involving 2708 patients with CHD and CHD risk equivalent undergoing treatment for dyslipidemia indicated that goal attainment in women was 25% less than the treatment goal attainment rate for men, after adjusting for other covariates [9].

Our study indicates that baseline LDL-C level is the main contributor to the gender disparity in lipid-lowering treatment goal attainment rate in China. It appears that the higher LDL-C level patients have before receiving treatment, the lower the goal attainment rate they will achieve after the treatment. Interestingly, previous studies have demonstrated that baseline LDL-C level is inversely associated with lipid-lowering goal attainment [13,14]. In the general population, women have higher LDL-C levels than men after 50 years of age, particularly in post-menopausal women [17]. Among the participants in our study, the average age of women was 65 years and approximately 90% of women were post-menopausal. The average baseline LDL-C level in women was 0.4 mmol/L higher than levels for men. This phenomenon might in part be explained by the presence of estrogen, which is associated with lowering cholesterol by reducing the LDL-C level through decreased LDL receptors [18] and an increased LDL clearance rate [19]. Reduced estrogens level in post-menopausal women attenuates the role of statins in reducing LDL-C level via mechanisms that increase LDL receptors and accelerate the metabolism of LDL. For this reason, women, particularly post-menopausal women, should focus more attention on their cholesterol levels and be given intensive treatment than men.

In our study, the dosage of statin alone accounted for a minor part of the gender disparity in lipid-lowering goal achievement rate. When we focused on high and very high risk patients, we found that women were less likely to be prescribed moderate or high doses of statins than men (59.6% vs 66.4%, *P* < 0.01). Statin therapy plays an important role in successfully attaining lipid-lowering goals [12], and higher goal attainment rates could have been achieved with higher doses of statin [20]. It has previously been shown that more women fail to achieve their lipid-lowering therapy partly due to the inadequate use of statin therapy [9]. This might be due to the incorrect perception of both female patients and their physicians regarding cardiovascular risk in women. A previous survey showed that physicians perceive women to be at lower risk than men, even if they had a similar calculated CHD risk [21] and led to the sub-optimal treatment of women with dyslipidemia. Improving awareness of CHD risk in women among physicians is an important step towards reducing the under-treatment with statin therapy in female patients.

The current study demonstrates that socioeconomic status appears to be a minor part of contributor to the gender disparity. In other studies, socioeconomic status was associated with the level of cholesterol and use of lipid-lowering drugs in patients, which is reflected in subject with lower levels of educational having higher levels of cholesterol [22,23], and lower income or medicare coverage was associated with poorer persistence with lipid-lowering therapy [24,25]. The L-TAP 1 survey found that highly educated participants were more likely to reach their LDL-C therapy goals than those with less education [12]. This led to the hypothesis that the gender disparity in LDL-C goal attainment rate may be partly attributableto gender differences in socioeconomic status, education level, occupation, and medical insurance coverage.

We also found that cardiovascular co-morbidities and associated risk factors accounted for the minority part of gender disparity in reaching lipid-lowering treatment goals. According to lipid treatment guidelines, the patients with high and very high CVD risk, combined with chronic diseases such as hypertension, CHD, DM and PAD, have more strict lipid-lowering treatment goals than patients who are at low risk of CVD. Reaching this goal will be a greater challenge for these high risk patients, especially for the women who were more likely to have co-morbidities in our study.

Our results have significant implications for clinical practice. Most of these factors are either modifiable or potentially amenable to interventions. At the policy level, possible interventions include enhancing the coverage rate of medical insurance and improving the healthcare environment for female patients. Patient-level interventions include reducing and controlling cardiovascular risk factors and improving compliance to lipid-lowering treatment. Physicians should promote an attitude of knowledge informing practice for the treatment of dyslipidemia in women, pay more attention to female patients who are postmenopausal and have high LDL-C levels,and provide an adequate and intensive treatment strategy to female patients.

The etiology influencing the lipid-lowering treatment goal attainment may be multi-faceted. In our study, all potential factors explained approximately one half of the gender disparity in lipid-lowering treatment goal attainment rate. The remaining half remains unexplained. It is possible that genetic factors may be among these unexplained factors. Studies on siblings and twins indicated that genetic factors can possibly explain approximately 70% of the effect of statin therapy [26]. For example, apolipoprotein E (ApoE) polymorphism could be the pharmacogenetic effect factor on statin therapy in patients with non-familial hypercholesteromia [27,28] and familial hypercholesteromia [29]. Furthermore, some studies indicate that the influence of ApoE polymorphism in respect to statin efficacy was different between women and men [30,31]. Gene-gender interaction may be a potential contributor to gender disparities in lipid-lowering treatment goal attainment. As far as other potential explanatory factors, more studies need to be done to discover them and elaborate on their effects in the future.

Despite having non-nationally representative samples, which is the main limitation of our study the results cannot be generalized beyond the study population, this survey provided benchmark data for interpretations of the gender disparity in lipid-lowering treatment goal attainment rate with large-scale nationwide samples. However, it should be noted that there may be some other factors which were not mentioned in our study that might influence the LDL-C treatment goal attainment such as the level of physical activity, psychological factors, family income, and others. Further studies should be conducted to confirm our findings and provide further insight. In addition, selective bias might lead to the gender-specified LDL-C treatment goal attainment. Previous data has shown that elderly women had lower outpatient rates and hospitalization rates than elderly men in China [32]. Therefore, our enrolled female patients might have more severe dyslipidemia compared with men. However, it is difficult to estimate the extent of any influence of selective bias on the gender disparity in treatment goal attainment rates.

## Conclusions

Understanding the factors and their contributions to the gender disparity in lipid-lowering treatment goal attainment rate is the first step to narrow the lipid-lowering treatment gap for women and men. In the current study, we performed logistic regression analyses to quantitatively evaluate the contributions of possible factors for gender disparity in lipid-lowering treatment goal attainment rate between women and men in hospitals across mainland China. The findings suggest that higher baseline LDL-C level, poorer socioeconomic status, more cardiovascular co-morbidities and associated risk factors, and lipid-lowing under-treatment account for approximately one half of the gender disparity observed in Chinese high and very high risk patients of dyslipidemia undergoing stable lipid-lowering therapy. But the other half of gender disparity remains unexplained. Further studies are needed to confirm our findings and explore the unexplained reasons for the gender disparity in lipid-lowering therapy goal attainment rate.

## Abbreviations

CHD: Coronary heart disease; PAD: Peripheral angiopathy disease; DM: Diabetes mellitus; LDL-C: Low-density lipoprotein cholesterol, HDL-C, high-density lipoprotein cholesterol; TC: Total cholesterol; TG: Triglycerides; OR: Odds ratio.

## Competing interests

The authors declare that they have no competing interests.

## Authors’ contributions

YW and LZ were in charge of the study design, questionnaire design, and quality control; GX and LL were in charge of the field investigation, quality control, and data arrangement; RZ was in charge of data arrangement, analysis, and paper writing. YW gave final approval of the manuscript to be published. All authors read and approved the final manuscript.
